# Spinal spatial integration of nociception and its functional role assessed via the nociceptive withdrawal reflex and psychophysical measures in healthy humans

**DOI:** 10.14814/phy2.14648

**Published:** 2020-11-20

**Authors:** Mauricio Carlos Henrich, Ken Steffen Frahm, Ole Kæseler Andersen

**Affiliations:** ^1^ Integrative Neuroscience Center for Neuroplasticity and Pain (CNAP) Department of Health Science and Technology Aalborg University Aalborg Ø Denmark

## Abstract

Animal studies have previously shown that deep dorsal horn neurons play a role in the processing of spatial characteristics of nociceptive information in mammals. Human studies have supported the role of the spinal neurons; however, the mechanisms involved, and its significance, remain to be clarified. The aim of this study was to investigate spatial aspects of the spinal integration of concurrent nociceptive electrical stimuli in healthy humans using the Nociceptive Withdrawal Reflex (NWR) as an objective indication of spinal nociceptive processing. Fifteen healthy volunteers participated in the study. Electrical stimuli were delivered, using five electrodes located across the sole of the foot in a mediolateral disposition, as a single or double simultaneous stimuli with varying Inter‐Electrode Distances (IEDs). The stimulation intensity was set at 1.5× NWR threshold (TA muscle). The size of the NWR was quantified in the 60–180 ms poststimulus window as a primary outcome measure. Psychophysical measures were secondary outcomes. Single stimulation elicited significantly smaller NWRs and perceived intensity than double stimulation (*p* < .01), suggesting the presence of spatial summation occurring within the spinal processing. During double stimulation, increasing the inter‐electrode distance produced significantly smaller NWR sizes (*p* < .05) but larger pain intensity ratings (*p* < .05). By the NWR, spatial summation was shown to affect the nociceptive processing within the spinal cord. The inhibited motor response obtained when simultaneously stimulating the medial and lateral side of the sole of the foot suggests the presence of an inhibitory mechanism with a functional, behaviorally oriented function.

## Graphical abstract

It is the first time that double simultaneous electrical stimulation is used to elicit the Nociceptive Withdrawal Reflex and investigate the spinal integration of nociception in healthy humans. Perceived intensities and reflex response were found to be differentially modulated by the inter‐electrode distance. A functional inhibitory spinal mechanism seems to be playing a relevant role in the elicitation of an optimal reflex, a model to explain the phenomenon is proposed.

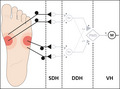

## 1 INTRODUCTION

Many pain‐related conditions are characterized by poorly localized pain areas in the body which is likely to reflect abnormal processing of spatial characteristics of the noxious phenomenon driving the condition (Gran, 2003; Graven‐Nielsen & Arendt‐Nielsen, 2010; Kamaleri et al., 2008; Wolfe et al., 1990). Within the spinal cord, several primary afferents converge onto spinal neurons and constitute the first relay for somatosensory integration. Electrophysiological studies in animals have shown that neurons located in this first relay play a role in encoding spatial aspects of the afferent input, including its location and intensity (Barber et al., 1978; Christensen and Perl, 1790; Kato et al., 2011; Price et al., 1978; Schouenborg, 2002; Schouenborg et al., 1994, 1995; Weng & Schouenborg, 1996). The translation of those findings into human studies remains a challenge since the direct assessment of neuronal activity in the human spinal cord is not possible. An indirect attempt has been made by comparing the electrophysiological assessment of dorsal horn neurons in rats with behavioral responses in healthy humans, presented to the same stimuli (Coghill et al., 1993a). That study suggested that dorsal horn neurons in humans may be coding spatial‐related features of nociceptive stimuli, such as its intensity, localization, and quality.

Behavioral studies in healthy humans have repeatedly observed phenomena such as spatial summation and lateral inhibition and have speculated about the mechanisms that may underlie these observations. Specifically, the presence of spatial summation on perceived intensities has been consistently confirmed for nociceptive stimuli of different natures, such as heat (Douglass et al., 1992; Nielsen & Arendt‐Nielsen, 1997; Price et al., 1989; Quevedo & Coghill, 2009; Staud et al., 2004), cold (Defrin et al., 2011), pressure (Defrin et al., 2003; Greenspan et al., 1997; Nie et al., 2009) and electrical stimulation (Reid et al., 2015). The neural mechanisms underlying spatial summation are based on simultaneous input to a certain postsynaptic neuron which integrates them to produce an increased net postsynaptic potential (Price et al., 1989). Stimulating a larger area will likely recruit a larger population of neurons, thus, projecting enhanced input to the postsynaptic neurons. Another mechanism that may underlie the spatial summation phenomenon is peripherally coded, stimulation of a larger proportion of the receptive field of a certain neuron positively contributes to its depolarization (Price et al., 1989). Moreover, it has been suggested that lateral inhibition may also be involved in the processing of nociceptive stimulation applied in a small skin area. An observation showing that a continuous thermal stimulus in the form of a moving line is perceived as less painful than two discrete points administered at the ends of that line (Quevedo et al., 2017), argues in favor of that hypothesis. In another study using noxious‐heat stimulation, the intensity of the perception was found to increase with the distance between stimuli, suggesting the presence of a lateral inhibition mechanism in the integration of nociceptive laser stimulation (Frahm et al., 2018), possibly covering smaller areas than spatial summation.

Most of the previously cited studies on the spatial integration of nociceptive stimuli in humans are exclusively based on psychophysical outcomes: pain intensity, stimulus localization, and pain quality. These measures arise from the processing of somatosensory information that went through, at least, three synaptic stages in the ascending pathway: dorsal horn of the spinal cord, thalamus, and somatosensory cortex. The NWR pathway, however, has a different projection compared to the ascending fibers within the spinal cord (Eccles and Lundberg, 1959; Schomburg, 1990). The reflex arc integrates afferent inputs across a well‐defined skin area to generate optimal withdrawal of the exposed area (Andersen, 2007; Kugelberg et al., 1960; Massé‐Alarie et al., 2019; Schouenborg & Kalliomäki, 1990; Schouenborg et al., 1994). As a large amount of evidence has been collected supporting the hypothesis that spinal networks likely contribute to the observations of spatial summation and lateral inhibition (Andersen et al., 1994; Coghill et al., 1993a, 1993b; Mørch et al., 2010; Price et al., 1989; Quevedo & Coghill, 2009; Quevedo et al., 2017; Wagman & Price, 1969), it would be highly interesting to apply a methodology to investigate these phenomena capable of assessing the spinal circuitry. Thus, the assessment of the Nociceptive Withdrawal Reflex (NWR) is an adequate technique to, in a noninvasive fashion, investigate the spinal spatial integration of simultaneous nociceptive stimuli. The use of the NWR is expected to provide complementary, objective evidence to the behavioral observations reported in previous studies through the psychophysical assessment of pain intensity and localization.

Particularly, it was hypothesized that due to the behavioral significance of the NWR, the neuronal processing and subsequent generation of a motor response is influenced by a spatial summation mechanism (double vs. single stimulation) within the spinal cord, provoking a stronger limb withdrawal when the area of stimulation increases, analogous to that reported in the literature for the perception of pain intensity. Using double stimulation with different inter‐electrode distances (IED), local lateral inhibitory mechanisms were also targeted and expected to be capable of inhibiting the motor response when two simultaneous stimuli were sufficiently close. Conversely, when the distance between the stimuli increases, mechanisms supporting spatial summation are expected to be facilitated and, therefore, reducing possible inhibitory effects due to lateral inhibition, leading to net larger NWRs. Secondary outcomes were also collected in an effort to obtain a psychophysical reference of the perceptual experience. Area‐ and distance‐based spatial summation of perceived intensities are also expected to be observed (Quevedo & Coghill, 2009; Reid et al., 2015). It is also predicted that the use of small diameter electrodes facilitates the stimulation of Aδ‐fibers which will lead to a sharp‐pricking perception quality (Beissner et al., 2010; Hugosdottir et al., 2019; Leandri et al., 2018; Mørch et al., 2011; Torebjörk & Hallin, 1973). Finally, increased IEDs are expected to enhance the spatial discrimination of double stimulation (Frahm et al., 2018; Mørch et al., 2010).

## 2 MATERIALS AND METHODS

### 2.1 Participants

Fifteen healthy volunteers, eight women and seven men, participated in the study (age 25 ± 5; mean ± *SD*). Subjects were excluded in case of pregnancy, breastfeeding, previous neural or mental disorders, disorders of the musculoskeletal system, inability to cooperate throughout the experimental session, presence of chronic or current acute pain (e.g., due to vigorous physical activity), pharmaceutical usage with known effects on nociception, or skin lesions on the sites of stimulation or recording. Subjects were given both oral and written instructions regarding the protocol prior to the experiment. Informed written consent was obtained from all participants. The study was approved by the local Ethical Committee (VN‐20180047) and was performed according to the Helsinki Declaration.

### 2.2 EMG recordings of the NWR

Three surface electrodes (Neuroline720, Ambu A/S, Denmark) were mounted on the skin over the Tibialis Anterior muscles following Surface EMG for the NonInvasive Assessment of Muscles (SENIAM) recommendations (Hermens et al., 1999). A reference electrode (Neuroline720, Ambu A/S, Denmark) was placed on the ipsilateral knee, over the patella. Surface EMG in double differential configuration was recorded (Frahm et al., 2012), amplified, bandpass filtered between 5 and 500 Hz, sampled at 2 kHz, stored, and analyzed offline. Custom‐made isolated amplifiers and software were used for EMG recordings (Jensen et al., 2015a).

### 2.3 Electrical stimulation

**FIGURE 1:**
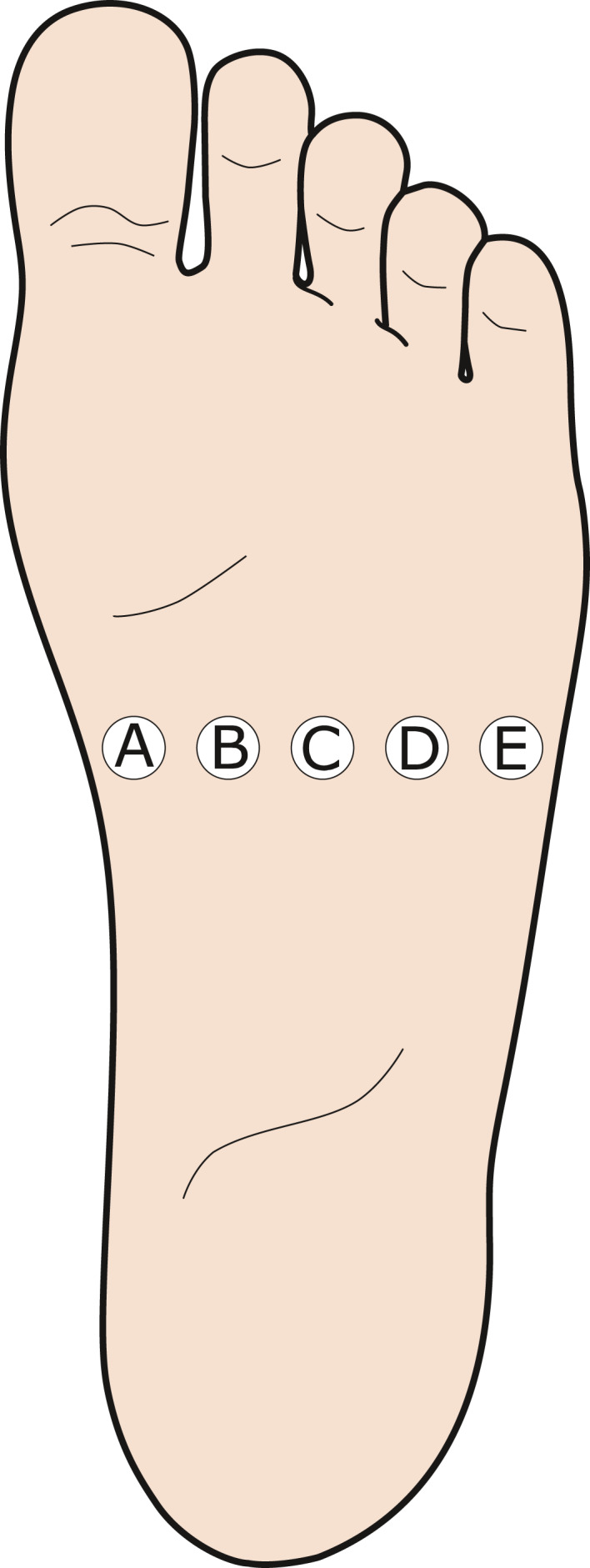
Configuration of the stimulation electrodes (A–E) mounted on the sole of the foot. A large anode was placed on the dorsum of the foot. Single electrical stimulation was delivered in each electrode and double stimuli in each pair of electrodes. The stimulation was always perceived in the sole of the foot

Five surface stimulation electrodes (Neuroline700, Ambu: A/S, Denmark) reduced to 28 square millimeters area (circular, diameter: 6 mm; to produce a very localized stimulus and to enable the mounting of five independent electrodes across the sole of the foot (Frahm et al., 2013)) were located on the sole of the foot over a mediolateral direction, above the tuberosity of the 5th metatarsal bone, as illustrated in Figure 1. Since the electrodes were evenly distributed, the inter‐electrode distance (IED) varied with the subject's foot size. One IED represented one‐fourth of the total width of the sole of the foot, typically equivalent to approximately 1.5 cm. By placing a large common anode (7.5 × 10 cm) on the dorsum of the foot, the stimulation was always perceived as applied on the sole of the foot. The stimulation electrodes were moved in case a radiating sensation was reported, indicating direct nerve activation. The stimulator output is directed to the relevant electrode using a custom‐made computer‐controlled relay (Jensen et al., 2015b; Neziri et al., 2009).

The stimulation parameters consisted of a 25 ms train composed of five 1 millisecond pulses delivered at 200 Hz (perceived as a single stimulus). The intensity was set at 1.5 times the NWR‐threshold (NWR‐t) for the five electrodes individually (detected in Tibialis Anterior muscle). The same intensity for each electrode was used for both single and double stimulation. When the stimulation intensity reached 50 mA or if the subject reported intolerable pain, the experiment was terminated. To prevent reflexes from habituating inter‐electrode intervals were randomized between 20 and 30 s (von Dincklage et al., 2013).

### 2.4 Estimation of the NWR threshold (NWR‐t) and NWR detection criteria

To determine the NWR‐t, an automated staircase protocol was used (Jensen et al., 2015b). Electrical impulses were delivered on each of the five stimulation electrodes in randomized order. The stimulation started with an initial intensity of 1 mA and was increased by steps of 2 mA until the first NWR was detected. Then, the intensity was decreased by 1 mA until the NWR was no longer detected. Thereafter, the intensity was increased and decreased in steps of 0.5 mA until a total of three descending and ascending limits were found. Finally, the NWR‐t was calculated by averaging the lasts two peaks and troughs.

Based on a previous study that compared methods for NWR detecting (Rhudy & France, 2007), the criteria to automatically detect the presence of an NWR was determined as an interval peak z‐score exceeding the numerical value of 12.

### 2.5 Experimental protocol

The study consisted of one experimental session divided into three blocks. During the first block (familiarization), a series of single electrical stimulations were applied in random order to the five electrodes (A–E) aiming at reducing the effects of arousal and anxiety.

During the second block, the stimulation intensity for each electrode position was defined. Thus, the NWR‐t was estimated for each of the five electrodes in random order. The stimulation intensity was defined by multiplying the obtained NWR‐t value by a fixed factor of 1.5 (see above).

Finally, electrical stimulations were applied in each of the five electrodes (single stimulation) and in each combination of electrode pairs (double stimulation). Seven repetitions of each condition were acquired. For every participant, all stimulations (single and double) were delivered in random order. Participants were lying comfortably in a reclined bed and instructed to avoid any voluntary muscle contraction. The EMG traces were continuously monitored in‐between stimuli to ensure that subjects did not make any voluntary contractions.

### 2.6 Outcomes

#### 2.6.1 NWR quantification

The NWR was quantified by calculating the root mean square (RMS) value of the EMG signal over the reflex window (60–180 ms poststimulus) (Andersen, 2007). Values were averaged across the seven repetitions, obtaining the average NWR size for every single and double stimulation. Data processing was performed off‐line using MATLAB software R2018b (Mathworks, Natick, MA, USA).

#### 2.6.2 Perceived intensity ratings

A Numerical Rating Scale (NRS) (anchored at 0 with “No perception,” at 5 with “Pain threshold,” and at 10 with “Worst pain imaginable”) was used to quantify the perceived intensity. After each stimulation, participants were asked to report the intensity of the perceived stimulation using the NRS. Values were averaged across the seven repetitions.

#### 2.6.3 Stimulus localization ability

After each stimulation, participants were asked to state which electrode(s) was(were) activated. A diagram, illustrated in Figure 1, was shown to the subjects to guide them to electrode positions. The frequency of correct reports was calculated. For single stimulation, a comparison between the different electrode positions was carried out. For double stimulation, the effect of the IED on the ability of the subject to discriminate a double stimulation (as coming from two independent sources) was assessed.

#### 2.6.4 Pain quality

The Short‐Form McGill Pain Questionnaire (SF‐MGPQ) was used as an exploratory outcome measure to observe the quality of the perception. Following the third stimulation of each condition, subjects were asked to indicate from a list of words that better characterized their perception. The list included the following descriptors: throbbing, shooting, stabbing, sharp, cramping, gnawing, hot‐burning, aching, heavy, tender, splitting, tiring‐exhausting, sickening, fearful, and punishing. The frequency that each of the words was selected for single and for double stimulation was calculated. The descriptors for intensities Mild, Moderate and Severe were collapsed into one group. Frequencies were averaged across the 15 subjects and normalized to the most frequently reported descriptor (Shooting, double stimulation, 53%). Word clouds were generated for the single and double stimulation condition in which the font size and color saturation of each descriptor are proportional to its normalized frequency.

### 2.7 Statistical analysis

Statistical analysis was performed using IBM SPSS 25. Data are presented as the mean and standard error. Prior to the analyses, data normality distribution was tested with a Shapiro–Wilk normality test. When normality was confirmed, RM‐ANOVA was performed. If a significant main effect was found, paired *t*‐tests between conditions were implemented and adjusted for multiple comparisons using the Bonferroni–Holm method. If the assumption of the normal distribution of the data could not be confirmed, Friedman's test was used. If found significant, Wilcoxon rank test was preferred for the pairwise comparisons with Bonferroni–Holm adjustment for multiple comparisons. *p* values .05 were considered significant.

## 3 RESULTS

### 3.1 NWR size

**FIGURE 2:**
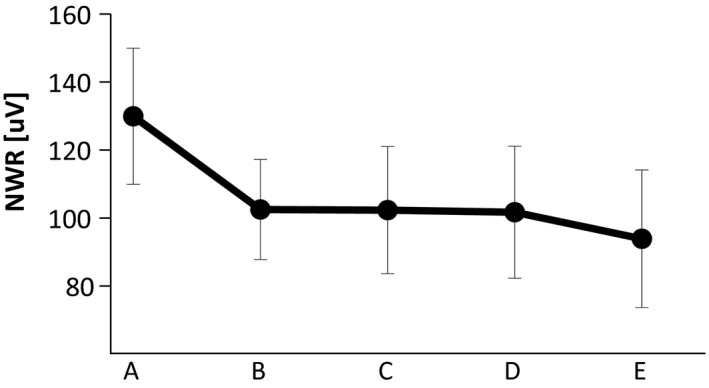
NWR sizes elicited by single stimulation. No significant differences in the size of the reflex were found between the stimulated sites. RM‐ANOVA, N.S

When comparing the size of the NWRs elicited by single stimulation (A–E, see Figure 1; stimulation intensities: 13.31 ± 1.06 [mA] (mean ± SE)), no significant differences were found related to the location of the stimulus (RM‐ANOVA: *F*(3,38) = 1.6, *p* > .05; Figure 2).

**FIGURE 3:**
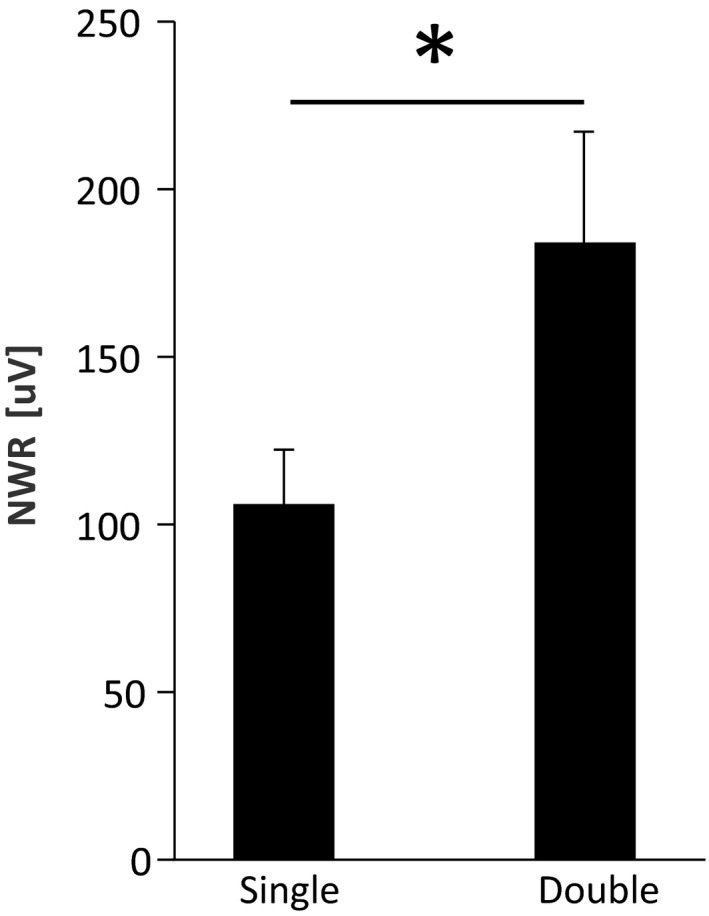
Averaged NWR for both Single (106 ± 16 µV) and Double (184 ± 33 µV) stimulation (mean ± SE) Double stimulation elicited significantly larger NWR than single stimulation. *: paired*t*‐test*p* < .01

The NWR size for double stimulation (184 ± 33 µV) was significantly larger than for single stimulation (106 ± 16 µV) (Paired *t*‐test, *p* < .01, Cohen's *d*_z_ = 0.83; Figure 3), likely suggesting a net effect of spatial summation on the NWR.

To compare the effect of the IED on the NWR size, the pairs with the same IED were pooled together for the analysis. This was considered acceptable since no differences were found between the NWR’ size elicited by the single stimulation at different sites (Figure 2). Moreover, the conditions that were pooled within each IED were compared and no differences were found between them (Friedman's test, *p* > .05).

**FIGURE 4:**
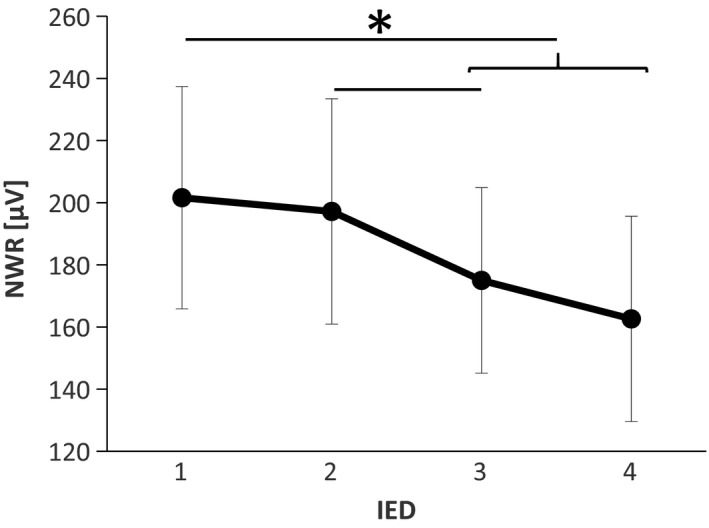
Reflex size (NWR) during double stimulation across different IEDs (mean ± *SE*). The main effect was found and, following adjusted post hoc comparison, NWR for IED = 1 was found to be significantly higher than IED = 3 and IED = 4, and IED = 2 higher than IED = 3. *: Friedman's test:*p* < .01 Post hoc: *p < .05

**FIGURE 5:**
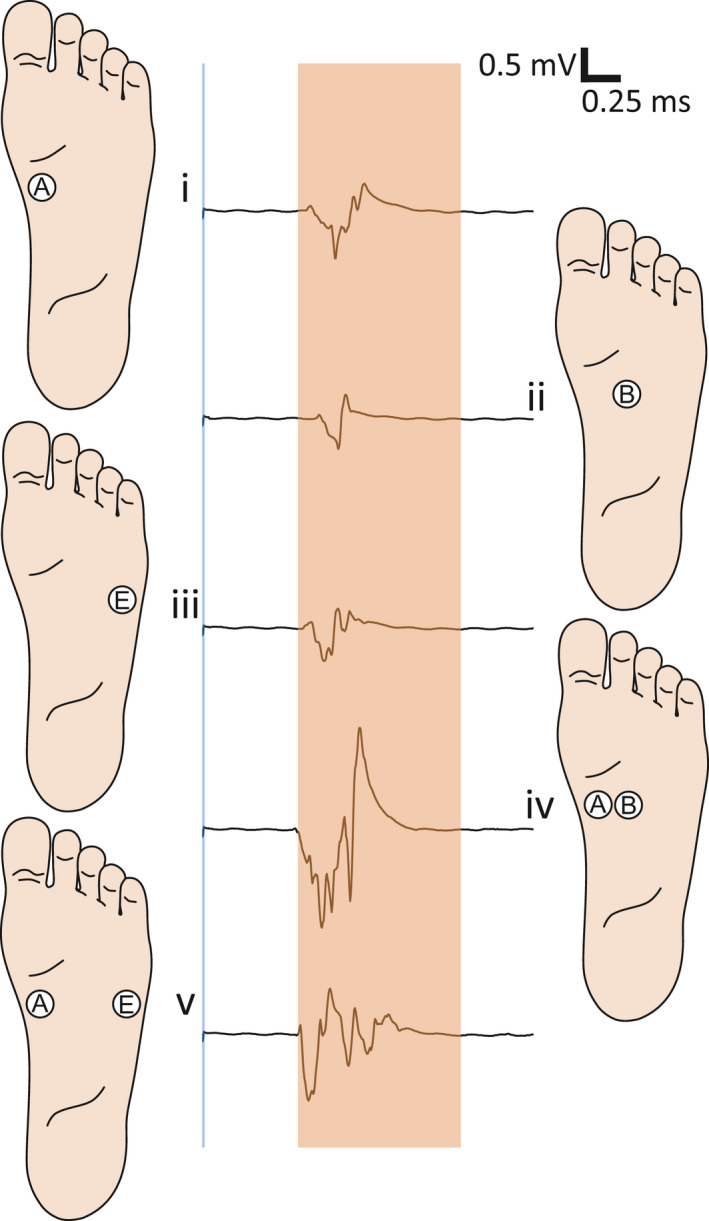
EMG traces of a representative subject on five different stimulation conditions: (i) Single stimulation in A; (ii) single stimulation in B; (iii) single stimulation in E; (iv) double simultaneous stimulation in A and B; and (v) double simultaneous stimulation in A and E

During double stimulation, a statistically significant difference was found in the NWR’ size depending on the IED (Friedman's test, *p* < .01; Kendal's *W* = 0.39) showing a tendency that larger IED resulted in smaller NWR values (Figure 4). Post hoc comparisons showed that the NWR elicited when using IED = 1 (202 ± 36 µV) was significantly higher than IED = 3 (175 ± 30 µV) (*p* < .01), and IED = 4 (163 ± 33 µV) (*p* < .01). Additionally, a significant difference was also found between IED = 2(197 ± 36 µV) versus IED = 3 (*p* < .05). See Figure 5 of EMG traces on a representative subject under different stimulation conditions.

### 3.2 Perceived intensity ratings

**FIGURE 6:**
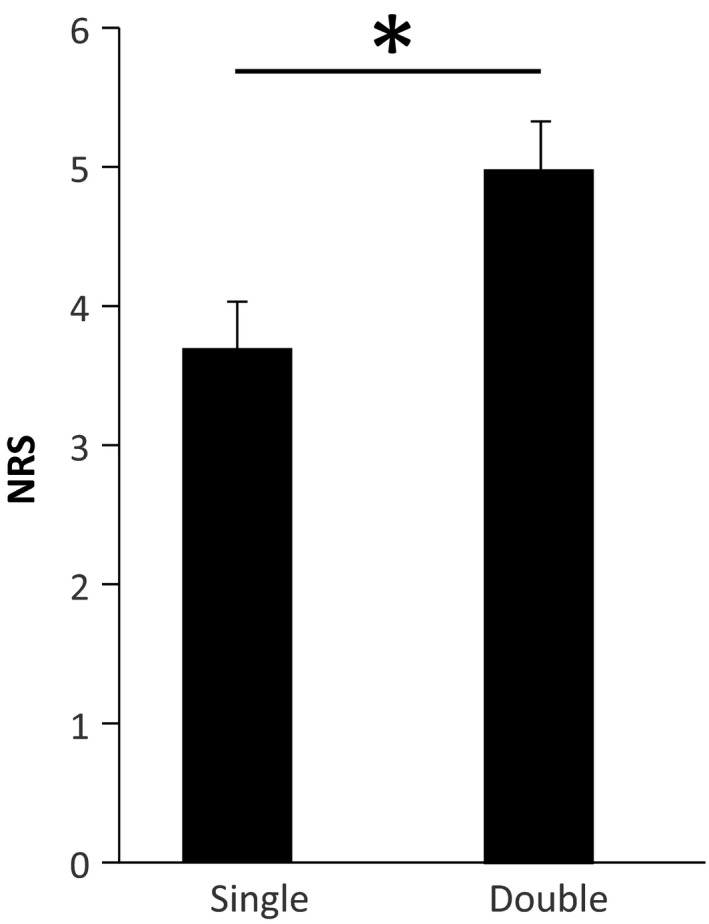
Averaged NRS during single (3.7 + 0.3) and double (5 ± 0.3) stimulation (mean ± *SE*). Double stimulation produced higher intensity ratings *: paired*t*‐test,*p* < .01

The reported perceived intensities (NRS) were significantly higher during double stimulation (5.0 ± 0.3) compared to single stimulation (3.7 + 0.3) (Paired *t*‐test, *p* < .01, Cohen's *d*_z_ = 2.5; Figure 6).

**FIGURE 7:**
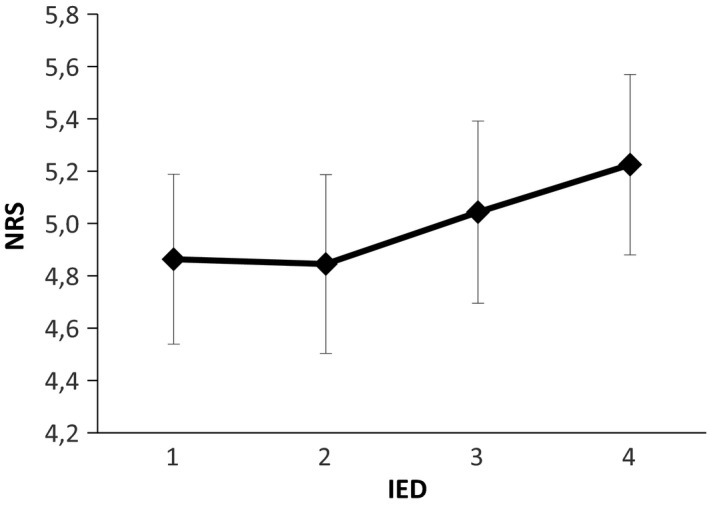
Perceived intensities during double stimulation across different IEDs (mean ± SE). A significant main effect was found (RM‐ANOVA:*p* < .05). Adjusted pairwise comparisons did not show significant differences between different IED

For double stimulation, a significant main effect of the IED was found (RM‐ANOVA: *F*(2,24) = 5 *p* < .05). However, adjusted pairwise comparison showed no statistically significant differences despite a tendency for larger NRS with increased IED (Figure 7).

### 3.3 Stimulus localization

The distribution of the perceived stimuli when delivering different stimulation combinations is summarized in Table 1. Each row represents the actual delivered stimulus and, on the columns, the location where the stimulation was perceived by the subject. Values are expressed as frequencies.

**TABLE 1:** Perceived stimuli localization. Each row represents the actual stimulated site(s). Columns represent the perceived stimulated site(s). For better visualization. Cells are formatted with a continuous color scale from green to red according to its value. Results are expressed as percentages (%). Sub.: Subtotal

		PERCEIVED STIMULATED SITE(S)																				
		A	B	C	D	E	Sub	A‐B	B‐C	C‐D	D‐E	Sub	A‐C	B‐D	C‐E	Sub	A‐D	B‐E	Sub	A‐E	Sub	Total
	Single stimulation																					
STIMULATED SITE(S)	A	71.4	14.3	1.9	0.0	0.0	87.6	11.4	0.0	0.0	0.0	11.4	1.0	0.0	0.0	1.0	0.0	0.0	0.0	0.0	0.0	100.0
	B	21.0	57.1	6.7	0.0	0.0	84.8	5.7	6.7	0.0	0.0	12.4	1.9	1.0	0.0	2.9	0.0	0.0	0.0	0.0	0.0	100.0
	C	0.0	15.2	61.9	9.5	1.0	87.6	0.0	8.6	1.9	0.0	10.5	0.0	1.9	0.0	1.9	0.0	0.0	0.0	0.0	0.0	100.0
	D	0.0	1.9	8.6	64.8	7.6	82.9	0.0	0.0	11.4	1.9	13.3	0.0	1.9	1.0	2.9	0.0	1.0	1.0	0.0	0.0	100.0
	E	1.0	0.0	1.9	36.2	46.7	85.7	0.0	1.0	1.9	7.6	10.5	0.0	0.0	3.8	3.8	0.0	0.0	0.0	0.0	0.0	100.0
	Double stimulation (IED = 1)																					
	A‐B	36.2	28.6	7.6	0.0	0.0	72.4	18.1	7.6	0.0	0.0	25.7	1.0	0.0	0.0	1.0	1.0	0.0	1.0	0.0	0.0	100.0
	B‐C	1.0	26.7	41.0	1.9	0.0	70.5	1.0	21.9	1.9	0.0	24.8	1.0	3.8	0.0	4.8	0.0	0.0	0.0	0.0	0.0	100.0
	C‐D	0.0	3.8	40.0	20.0	1.9	65.7	0.0	6.7	17.1	4.8	28.6	0.0	3.8	1.0	4.8	1.0	0.0	1.0	0.0	0.0	100.0
	D‐E	0.0	0.0	6.7	34.3	23.8	64.8	0.0	1.0	14.3	13.3	28.6	0.0	3.8	1.9	5.7	0.0	1.0	1.0	0.0	0.0	100.0
	Double stimulation (IED = 2)																					
	A‐C	11.4	29.5	14.3	1.9	0.0	57.1	11.4	22.9	0.0	0.0	34.3	5.7	2.9	0.0	8.6	0.0	0.0	0.0	0.0	0.0	100.0
	B‐D	0.0	6.7	41.9	17.1	6.7	72.4	0.0	6.7	12.4	1.0	20.0	0.0	6.7	1.0	7.6	0.0	0.0	0.0	0.0	0.0	100.0
	C‐E	0.0	1.0	21.0	29.5	15.2	66.7	0.0	1.0	22.9	4.8	28.6	0.0	1.9	1.9	3.8	0.0	1.0	1.0	0.0	0.0	100.0
	Double stimulation (IED = 3)																					
	A‐D	1.0	9.5	21.0	8.6	3.8	43.8	7.6	11.4	10.5	1.0	30.5	3.8	7.6	1.0	12.4	11.4	1.0	12.4	1.0	1.0	100.0
	B‐E	1.0	1.0	14.3	28.6	18.1	62.9	0.0	4.8	9.5	7.6	21.9	1.0	7.6	1.0	9.5	1.0	4.8	5.7	0.0	0.0	100.0
	Double stimulation (IED = 4)																					
	A‐E	0.0	2.9	8.6	21.0	9.5	41.9	5.7	12.4	11.4	4.8	34.3	1.0	5.7	1.9	8.6	5.7	4.8	10.5	4.8	4.8	100.0

During single stimulation, the stimulated location was more often perceived correct when stimulating the medial electrodes than the lateral electrodes; however, the differences were not found to be statistically significant (Friedman's test, *p* > .05) (Table 1).

**FIGURE 8:**
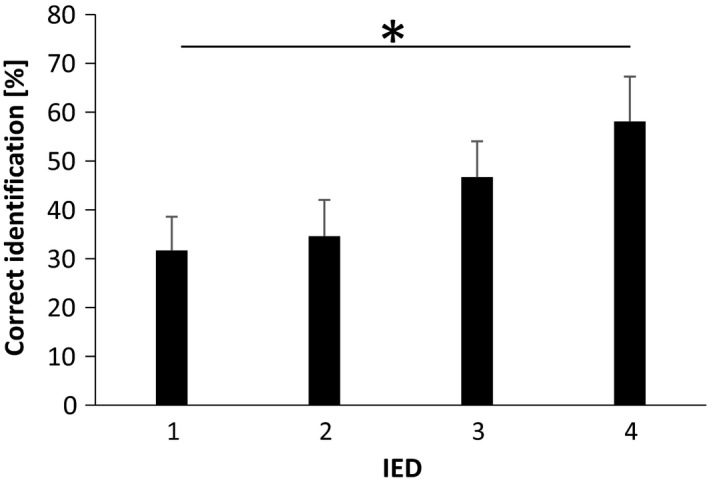
Percentage of correct identification during double stimulation. The frequency of correct identification Increased with IED (Friedman's test:*p* < .05; Post hoc:*p* < .01)

When assessing the effect of the IED on the ability of the subject to correctly identify double stimulation, a statistically significant main effect was found (Friedman's test, *p* < .05). Post hoc analysis, showed statistically significant larger percentage of correct identification for IED = 4 compared to IED = 1 (*p* < .01) (Figure 8).

### 3.4 Pain quality

**FIGURE 9:**
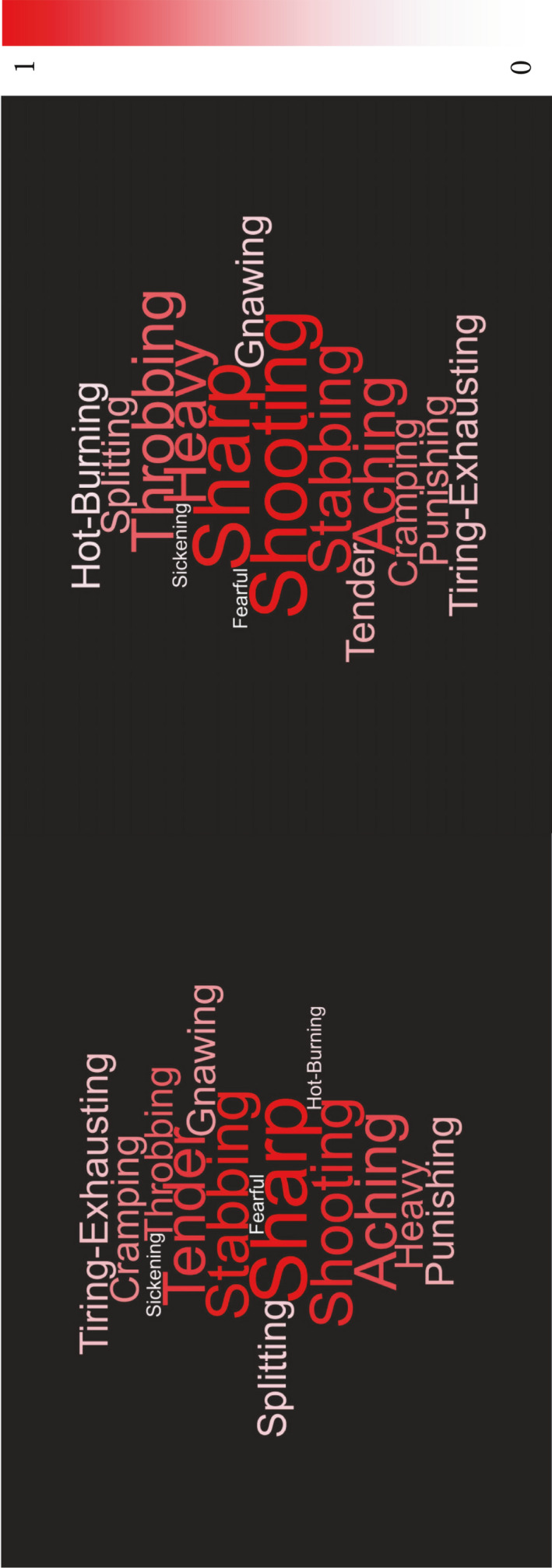
Word clouds generated for the pain quality observation when using single stimulation (A) and double stimulation (B). The normalized frequency is coded by the font’ size and the color saturation of each descriptor

The descriptors most frequently chosen to describe the perception quality were *Shooting*, *Stabbing*, *Sharp*, *Aching*, *Heavy,* and *Tender*. For single stimulation, the most frequent quality reported was *Sharp* (48%), followed by *Stabbing* (31%), *Shooting* (29%), and *Tender* (21%). Moreover, when delivering a double stimulation, the descriptor most frequently chosen was *Shooting* (53%), followed by *Sharp* (47%), *Stabbing* (41%) and *Aching* (29%) (Figure 9).

## 4 DISCUSSION

This is the first study in which simultaneous electrical stimuli have been used to elicit an NWR and to investigate how spatial integrative mechanisms may affect the processing of nociceptive stimuli. The administration of double stimuli (compared to single) resulted in enhanced responses, both seen as increased intensity ratings (as previously reported Mørch et al., 2010; Reid et al., 2015)) but also a larger motor response, arguing for the presence of spatial summation phenomenon at the spinal level. During double stimulation, the NWR size and the perceived intensities were modulated by the IED, in opposite directions. Interestingly, the modulation of the NWR showed inhibited responses for larger IED, suggesting the presence of a spatial inhibitory mechanism with a functional role in agreement with the spinal intrinsic reflex organization.

### 4.1 Spinal integration during double stimulation

TA NWR elicited by single stimulation were not different across the sole of the foot (Figure 2), as it was expected since the intensity of the stimuli was defined as a multiple of the NWR‐t for each stimulation electrode (Figure 1). This means that in the conditions of this experiment when stimulated individually, there was no effect of the spatial location of the stimulus in the NWR.

It is the first time that two simultaneous electrical stimuli applied in a small skin area are used to elicit the NWR in humans. Our results showed that double stimulation resulted in larger NWRs (Figure 3) than single stimulation, supporting that spatial integration is a key mechanism in the processing of nociceptive input at the spinal level. The exact neural basis of these spinal mechanisms remains challenging to address in a direct fashion since invasive electrophysiological techniques are obviously not possible in humans. However, the present methods of indirect evidence, complemented by human imaging studies and animal studies (see (Cordero‐Erausquin et al., 2016) for a review), suggest that the dorsal horn of the spinal cord are integrating afferent spatial information.

In this study, the use of double simultaneous stimulation with different IED enabled us to investigate the spinal nociceptive system in relation to spatial aspects of nociceptive integration and further interpreting the critical defensive role of the NWR in the perspective of the modular reflex organization (Andersen, 2007; Andersen et al., 1999; Julious, 2004; Levinsson et al., 1999; Massé‐Alarie et al., 2019; Schouenborg et al., 1994; Sonnenborg et al., 2000).

The present findings do not support the hypotheses that a locally mediated lateral inhibition mechanism cause a reduction in the NWR size when two stimuli are delivered in close proximity as it was reported for perceived intensities in other studies (Frahm et al., 2018; Quevedo & Coghill, 2009; Reid et al., 2015), even when using the smallest inter‐electrode distance (Figure 4). Conversely, when increasing the IED (particularly for IED = 3 and 4), the elicited NWRs were smaller. The observed reflex sizes represent the net results of the interaction between mechanisms of summation and inhibition, the specific contribution of a locally mediated lateral inhibitory effect, if present, is likely masked by a stronger facilitatory effect. A plausible explanation for this phenomenon could be based on the modular organization of the NWR (Andersen, 2007; Andersen et al., 1999; Schouenborg et al., 1994; Sonnenborg et al., 2000). According to this model, each muscle involved in the NWR has its own reflex receptive field (RRF). The reflex receptive field of the TA muscle is located medially, and distal on the arch of the foot and stimulation herein triggers TA contraction producing dorsal flexion and inversion of the foot (Andersen et al., 1999, 2001; Grimby, 1963; Neziri et al., 2009). Moreover, the stimulation of the lateral side of the sole of the foot evokes dorsal flexion and eversion of the foot (Andersen et al., 1999). Focusing on the inversion and eversion in the talocalcaneal joint, contraction of TA only serves to generate inversion, while eversion is mainly generated by the peroneus longus muscle (Andersen et al., 1999). Particularly for the IED = 3 and IED = 4 (Figure 4), the two stimuli were applied simultaneously in the arch and the lateral side of the sole of the foot (Figure 1), meaning that reflex the activation of muscles generating inversion and eversion would be concurrently evoked with no functional purpose.

Animal studies and human reflex studies support that the modular organization of the NWR may partially explain the inhibitory phenomenon observed in this study, that is, decreased NWR size for increasing IEDs (Figures 4, 5 and 5). Specifically, a study performed in decerebrated spinal rats directly assessed the reflex receptive field spatial organization and argued in favor of the existence of an inhibitory mechanism between reflex pathways (Weng & Schouenborg, 1996) That study showed that the application of a conditioning stimulus to the rats’ hind paw inhibits activity in muscles which recruitment would move the ipsilateral limb toward the stimulus. These results strongly suggest that dynamic inhibition between reflex pathways do exist, at least in animals, and serves functional purposes. Human studies have also provided indirect evidence supporting the existence of an inhibitory phenomenon between reflex modules. Notably, a reflex study performed in healthy humans has shown the inhibition of voluntarily contracted muscles when a stimulus is applied in its inhibitory receptive field (Sonnenborg et al., 2000). Other studies assessing the NWR during rhythmic muscle contraction (e.g., gait) further support the existence of an inhibitory phenomenon between reflex receptive fields of nonsynergistic muscles in healthy humans (Richard et al., 2015).

**FIGURE 10:**
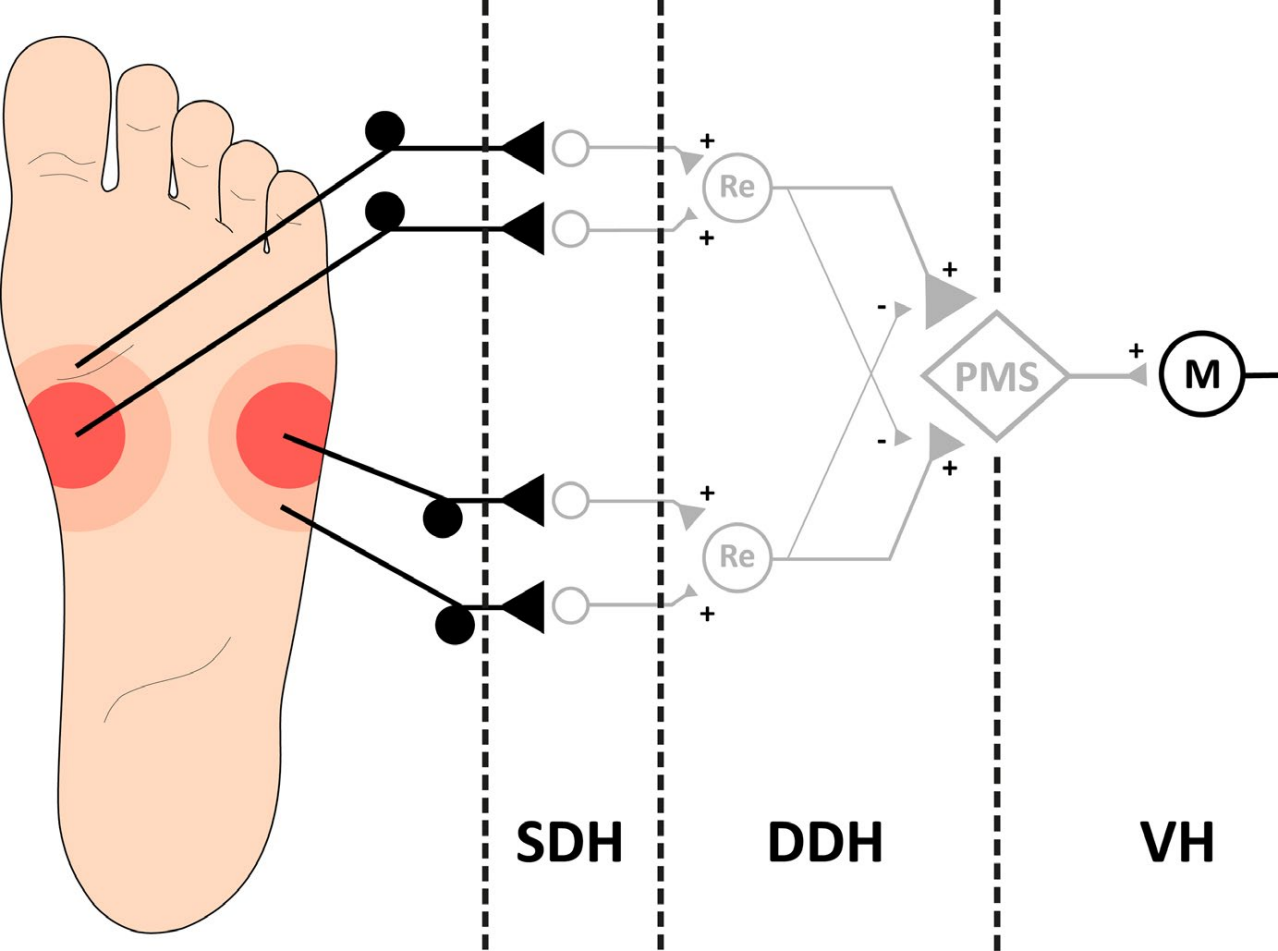
A schematic of the model depicting the functional organization of the NWR circuitry together with the mutual inhibition of different RRF proposed in this study, that may explain the results obtained. The size of the synaptic terminals represents the weighing effect of the reflex encoders (Re) and the premotor system (PMS) related to the withdrawal efficacy of the muscle that is recruited (Schouenborg, 2002). Double simultaneous stimulation of the medial and lateral side of the sole of the foot produced an inhibited motor response in TA. Mutual inhibition between RRFs is represented by lateral inhibitory projection on Re. Descending modulatory fibers are not shown although they are thought to affect the NWR pathway by acting pre and/or postsynaptically on the interneurons (shown in gray) and/or on the Re in the dorsal horn (Biurrun Manresa et al., 2014). The potential descending modulatory effect produced by those fibers was minimized in this study as explained in the discussion section. The output of the PMS is transformed by the alpha motor neurons (M) into the recruitment of the optimal muscle or group of muscles to withdraw the limb from the stimuli. Neurons colored in gray conceptualize the net synaptic effect and do not represent a single direct connection in the human spinal circuitry. SDH: Superficial Dorsal Horn; DDH: Deep Dorsal Horn; VH: Ventral Horn

From a behavioral point of view, the preferential recruitment of the TA would not anymore represent a favorable solution to efficiently withdraw the limb from the potentially harmful stimulus. To produce the optimal response to this stimulus (IED = 3 and IED = 4; Figure 4), a more complex spinal integration might be needed, and the withdrawal might involve proximal muscles. Reduced activation of the TA together with the recruitment of muscles subserving the eversion would lead to a net stabilization in the talocalcaneal joint which seems to be the optimal withdrawal strategy for the presented complex nociceptive input. Thus, the observed TA reflex modulation (Figure 4) appears to have a functional role in accordance with the modular organization of the NWR. The muscle recruitment strategy seems to be specific to the spatial characteristics of the stimulus. An inhibitory mechanism, acting on lateral reflex receptive fields, as the one described in other somatosensory systems (Békésy, 1962; Coren, 1970), seems to be essential for this task, since it would enhance the spatial discrimination and consequentially, allow the generation of a more efficient protective response. For this behavioral oriented system, the concept of laterality would not be defined by the adjacency of the RF of the primary afferents (as it is in visual, tactile senses (Bekesy, 1967)), but most probably by the reflex receptive field encoded by deeper neurons in the spinal cord. The presence of a functional lateral inhibition mechanism playing a role in the deep dorsal horn along with the encoding of the reflex receptive field might be the mechanism by which adjacent reflex receptive fields exert mutual inhibition when several reflex receptive fields are activated concurrently (Figure 10). Figure 10 depicts a schematic model of the organization of NWR circuitry, including the inhibitory projections that may explain the results of this study when two stimuli are simultaneously applied in the medial and lateral sole of the foot. Additionally, this model also accounts for the functional organization of the NWR elicited by single stimulation, as shown in previous animal and human studies (Biurrun Manresa et al., 2014; Schouenborg, 2002).

However, it should be noted that supraspinal descending inhibition may also be triggered in studies in which participants are instructed to contract the muscles previous to the stimulation. The instruction to perform the action may serve as an attentional cue that a painful stimulus is imminent and therefore affecting the NWR (Bjerre et al., 2011). Top‐down modulatory pathways are known to modulate the overall spinal nociceptive processing (Bartolo et al., 2013; Biurrun Manresa et al., 2014; Bjerre et al., 2011; Cleland & Bauer, 2002; Harris & Clarke, 2003; Levinsson et al., 1999; Roy et al., 2009; Schwindt, 1981; Villemure & Bushnell, 2002). In the present study, however, by randomizing the delivered stimuli, supraspinal modulation is not expected to differentially affect any particular condition. In any case, if a supraspinal control is exerted over all the conditions, its effect would be canceled out in the comparison. Additionally, the reflex was quantified in a 60–180 ms poststimulus interval which should exclude the potential effect of cortical structures. It is unlikely that in such a short time window, superior structures could cause a differential descending drive based on the spatial characteristics of the individual stimulus, implying that the behavioral‐functional response is coordinated by spinal structures (Andersen, 2007; Hugon, 1973; Sandrini et al., 2005).

### 4.2 Psychophysical outcomes

#### 4.2.1 Perceived intensity

The larger perceived intensity ratings during double stimulation compared to single (Figure 6), are most likely due to Spatial Summation (Nie et al., 2009; Reid et al., 2015; Staud et al., 2004). As stated above, the exact mechanisms are still not clearly described; however, previous evidence suggested that it may be due to two different processes: neuronal recruitment and local integration (Price et al., 1989). The argument for the former is that when increasing the area of stimulation (or the distance between two simultaneous stimuli) the probability of recruiting a larger population of neurons increases and therefore, the perceived intensity also increases. Moreover, local integration refers to the ability of a nociceptive neuron to summate different inputs applied in its own receptive field (RF). When comparing single versus double stimulation these two mechanisms may coexist explaining the perceived intensity results obtained in this and other studies.

The size of the RFs of the primary nociceptive afferents in the sole of the foot has not been studied in humans; however, some studies have reported the RF size of nociceptive neurons in the dorsum of the foot rarely cover a longitudinal distance larger than 2 cm (Schmidt et al., 1997; Torebjork et al., 1984; Torebjork, 1974). In our experiment, for IED = 3 and IED = 4, the inter‐electrode distance ranges approximately from 4 to 6 cm. The probability of stimulating different and not‐overlapping RFs increase, and therefore a larger neuronal population is more likely to be recruited. This could explain why the reported intensities were larger in those conditions (although not significant in the corrected multiple comparisons, Figure 7), also supporting the hypotheses that the process of spatial summation may take place by integration in the deep dorsal horn or further up the neuroaxis.

Lower perceived intensity ratings for smaller IEDs may be explained by the presence of a local lateral inhibition mechanism being facilitated when two stimuli are applied close to each other, reducing the net output reaching superior structures. In agreement with the present findings, another study reported that for an IED of 0 cm, (simultaneous double stimulation applied near each other) likely favoring the recruitment of a locally mediated lateral inhibitory mechanism, the spatial summation was not statistically significant (Reid et al., 2015). The presence and relevance of the lateral inhibition mechanism in the perceived intensities were previously suggested in (Frahm et al., 2018) and (Quevedo et al., 2017), although in these studies researchers used laser stimulation in form of lines versus discrete points. Assuming the same mechanisms are recruited in the present experiment with electrical stimulation, line stimulation can be considered as several discrete points applied very close to each other, and because the IEDs used in those studies resembles ours, lateral inhibition appears to be playing an important role in nociceptive processing for encoding location and intensity and could explain part of our results.

#### 4.2.2 Localization and perceived qualities

As the aim was to study the spinal integration of multiple stimuli using the NWR, the stimulation intensity was determined based on the NWR threshold and stimulation intensities were higher toward the lateral part of the sole of the foot. Although this may affect the localization ability of the subjects (Steenbergen et al., 2014), in the present experiment, for single stimulation, no significant differences were found in the ability of the subject to localize a single stimulation due to the position of the electrode on the sole of the foot (Table 1). The ability to discriminate double stimulation as being two independent stimuli has been shown to depend on the stimulation intensity (Steenbergen et al., 2014), and on the region of the body stimulated (probably due to differences in innervation density, skin thickness, and somatotopic organization in the brain) (Kowalzik et al., 1996; Mancini et al., 2015; Mørch et al., 2010; Weissman‐Fogel et al., 2012), and on the IED (Defrin et al., 2006, 2011; Frahm et al., 2018; Mancini et al., 2015; Price et al., 1989; Weissman‐Fogel et al., 2012). In the present study, the ability of the subjects to perceive two independent stimuli when delivering a double stimulation was assessed by asking the subject to identify the activated electrode(s). A statistically significant effect in relation to the IED was found (Friedman's test, *p* < .01; Figure 8), showing that subjects could discriminate easier the two stimuli when they were separated by approximately 6 cm (IED = 4) than when they were closer to each other (IED = 1). A factor that seems to be affecting the spatial resolution in the perception of two independent stimuli, which might explain why smaller IED could not be easily discriminated, is the overall large skin thickness of the sole of the foot. Although not significant, the skewness observed toward the medial electrodes (Table 1) suggests that the effect of the skin thickness modifies the electrical field during stimulation and thus affecting the activation of the afferent fibers (Frahm et al., 2013).

Last, regarding the observed quality of the perception, the descriptor most commonly chosen for single stimulation was *Sharp,* while for double stimulation, the most frequently selected descriptor was *Stabbing* (Figure 9). For both types of stimulations, the use of small diameter electrodes appeared to evoke a sensation indicative of high proportion of Aδ‐fiber activation, as it was expected (Beissner et al., 2010; Frahm et al., 2013; Hugosdottir et al., 2019; Mørch et al., 2011).

In summary, regarding the perception of the stimuli, the presence of a lateral inhibition mechanism in balance with spatial summation, may explain the results obtained in this study. Spatial summation was found to play a similar defensive role in both the NWR and the overall perceived intensity of the stimulus. However, when the IED increased, the NWR and the perceived intensities were modulated in opposite fashions (the size of the NWR decreased while the stimulus was perceived as more intense). Double simultaneous stimuli applied in the medial and lateral sides of the sole of the foot, elicited an inhibited motor response (compared to smaller IEDs). This modulation suggests the presence of an inhibitory spinal mechanism, with a functional organization, acting upon laterally located reflex receptive fields. This mechanism allows an optimal withdrawal response specifically related to the spatial characteristics of the stimulus and the stimulated parts in the body. The differential modulation observed between the NWR and the perceived intensities, seem to be the result of two systems that do not appear to be linked. A functional inhibitory mechanism between reflex receptive field encoders in the pathway from deep dorsal horn to the spinal motor output may explain the NWR results. Since those reflex encoders do not have ascending collaterals (Andersen, 2007; Schouenborg et al., 1994), their interaction would be transparent to supraspinal structures integrating the intensity of the perception. Additionally, fibers coding pain intensity are likely diverging earlier in the pathway to produce the ulterior perception of pain. These may be explaining the difference observed in the effect of the IED between the NWR and the perceived intensities (Figures 4, 7 and 7).

Future studies are needed to investigate the NWR from several muscles during simultaneous stimulation, both in the ipsilateral and contralateral limb to thoroughly assess the proposed model. Autonomic responses (such as heart rate or skin conductance) could also have been considered in the present study to further assure that descending effects were not affecting the results. However, since the stimulation conditions were totally randomized, the probability of a condition‐specific modulation is minimized.

## CONFLICT OF INTEREST

There are no conflicts of interest.

## AUTHOR CONTRIBUTION

All authors conceived and designed the study; M.C.H. performed the experiments and analyzed the data; all authors interpreted the results of the experiments, drafted, revised, and approved the final version of the manuscript.

## ETHICAL **STATEMENT**

The study was approved by the local Ethical Committee (VN‐20180047) and was performed in accordance with the Helsinki Declaration. All participants provided written informed consent.

## Data Availability

Datasets are available from the corresponding author on a reasonable request.
